# Huperzine A protects against traumatic brain injury through anti-oxidative effects via the Nrf2-ARE pathway

**DOI:** 10.22038/IJBMS.2021.58169.12932

**Published:** 2021-10

**Authors:** Zhengrong Mei, Ye Hong, Haiyi Yang, Qiongyu Sheng, Bing Situ

**Affiliations:** 1 Department of Pharmacy, Key Laboratory for Major Obstetric Diseases of Guangdong Province, The Third Affiliated Hospital of Guang-zhou Medical University, Guangzhou, Guangdong Province, 510150, P.R. China; 2 Guangzhou Medical University, The Third Affiliated Hospital of Guangzhou Medical University, Guangzhou, Guangdong Province, 510150, P.R. China; 3 Department of Pharmacy, Key Laboratory for Major Obstetric Diseases of Guangdong Province, The Third Affiliated Hospital of Guangzhou Medical University, Guangzhou, Guangdong Province, 510150, P.R. China

**Keywords:** Huperzine A, Neuroprotection, Nrf2-ARE, Oxidative stress, Traumatic brain injuries

## Abstract

**Objective(s):**

Traumatic brain injury (TBI) is a prominent health problem worldwide and it may lead to cognitive dysfunction, disability, and even death. To date, there is no effective treatment for TBI. Our previous study showed that Huperzine A (HupA) improved cognitive function in a mouse model of TBI. However, the detailed mechanism of HupA remains unaddressed. In this study, we investigated the possible mechanism of the neuroprotective effect of HupA.

**Materials and Methods:**

C57BL/6 mice were randomly divided into 3 groups as sham, injured with vehicle treatment, and injured with HupA treatment groups. The Morris water maze task was used to evaluate the impairment of special learning and memory. Brain edema was as-sessed by measuring the wet weight to dry weight ratio. Malondialdehyde (MDA) and glutathione peroxidase (GPx) levels were measured for oxidative stress. Protein expressions of nuclear factor erythroid 2-related factor 2 (Nrf2), heme oxygen-ase-1(HO-1), and synaptophysin were detected by Western blot. The brain sections were stained with hematoxylin-eosin (H&E) for histology study.

**Results:**

We found that HupA therapy improved histology and cognitive functional outcomes after TBI. HupA reduced brain edema in TBI mice. furthermore, HupA inhibited ox-idative stress. HupA promoted nuclear factor erythroid 2-related factor 2 (Nrf2) nu-clear translocation and activated Nrf2 after TBI.

**Conclusion:**

HupA protects against TBI through antioxidative effects via the Nrf2-ARE pathway.

## Introduction

Traumatic brain injury (TBI) is a leading cause of death and disability in young and aged populations and it has been a major public health problem in the modern society (1). The pathophysiology of TBI includes primary and secondary brain injuries. Primary brain injury occurs at the time of trauma. Common mechanisms include direct impact, rapid deceleration, blast waves, and penetrating injury. Although primary brain injury is the major factor determining the main outcomes, the secondary brain injury may further exacerbate the damage of TBI (2, 3). Secondary brain injury after TBI is usually considered a molecular cascade that begins at the time of initial trauma and continues for hours or days. The pathophysiology of secondary brain injury is poorly understood. Several factors including oxidative stress, excitotoxicity, apoptosis, mitochondrial dysfunction, and inflammatory responses contribute to its development.

Oxidative stress has been implicated in many disease processes including TBI and other neurodegenerative disorders. Nuclear factor erythroid 2-related factor 2 (Nrf2) is an important transcription factor that regulates the cellular oxidative stress responses and protects against a variety of harmful stresses (4). Nrf2 activates the anti-oxidant response and induces a wide range of gene transcriptions to counteract the harmful effects of oxidative stress and restore intracellular homeostasis (5). Under normal conditions, Nrf2 is located in the cytoplasm and binds to Kelch-like ECH-associated protein (Keap1), which blocks Nrf2 from entering the nucleus (6-8). When oxidative stress occurs, Nrf2 translocates from the cytoplasm to the nucleus, resulting in the increased anti-oxidant enzymes and decreased oxidative damage (4). Nrf2 is activated in TBI and is considered an endogenous compensatory adaptation against TBI-related oxidative stress (9).

TBI causes significant mortality and complications. Though several pathways have been the focus of preclinical work to develop treatments for preventing secondary brain injury after TBI, there is no effective treatment so far. Anti-oxidants suppress oxidative stress via intracellular ROS scavenging. Therefore, anti-oxidant strategies are of particular interest in the prevention of ROS-induced neuronal dysfunction and degeneration after TBI. Huperzine-A (HupA), a natural sesquiterpene alkaloid from the fir moss *Huperzia Serrata*, is a reversible and selective acetylcholinesterase (AChE) inhibitor (10, 11). Moreover, HupA has shown anti-inflammatory, neuroprotective, and anti-oxidant properties in various neurodegenerative diseases (12). 

HupA has demonstrated neuroprotective effect, improvement of cognitive functions, and slowing the progression of Alzheimer’s Disease (AD) in patients (13-16). It is currently being investigated as a possible treatment for neurodegeneration and AD. Furthermore, previous work from our laboratory showed that treatment with HupA inhibited oxidative stress, reduced inflammation, and improved behavioral outcomes in a TBI animal model (17). In this study, we further investigated whether HupA affected oxidative stress in a TBI model to better its potential therapeutic mechanisms. Our results showed that HupA reduced the TBI-induced oxidative stress in the brain by activating the Nrf2 pathway, inhibiting heme oxygenase-1(HO-1) protein expression. 

## Materials and Methods

All experiments were approved by the Third Affiliated Hospital of Guangzhou Medical University Institutional Animal Care and Use Committee and complied with the National Institutes of Health (NIH) Guide for the Care and Use of Laboratory Animals.

***Mouse model of closed head injury (CHI)***

C57BL/6 male mice (6 weeks old) were purchased from Guangdong Medical Laboratory Animal Center. The repetitive mild closed head injury (CHI) model was used as previously described (18) with some modifications. Briefly, C57BL/6 male mice were anesthetized for 45 sec using 3% isoflurane in a 70:30 nitrous oxide/oxygen mixture. Anesthetized mice were placed on a delicate task wiper and positioned such that the head was placed directly under a hollow guide tube. A 54g metal bolt was dropped from a 28 inches height tube on the dorsal aspect of the skull. Injured mice underwent 7 concussive injuries over 9 days. Mice were injured daily for 5 days, followed by 2 days without injury, and then by two additional daily injuries. Sham injury mice were subjected to anesthesia without trauma. After injury, mice were returned to their home cages, with food and water.

***Experimental protocol***

**Figure 1:**
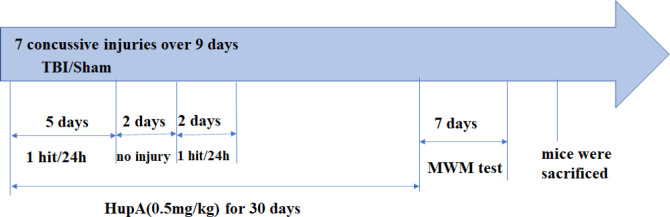
Study design scheme. Figures show drug treatment and experimental design

TBI: Traumatic brain injury; MWM: Morris water maze

Mice were randomly divided into 3 groups: sham (n =20), TBI with vehicle treatment (n =20), and TBI with HupA treatment (n =20). The TBI + HupA group were subjected to brain injury then were administered daily intraperitoneally (IP) injection of HupA. The dose of HupA was selected by targeting clinically significant concentrations as described previously (19). HupA (98% pure; Sigma-Aldrich, St. Louis, MO, USA) was dissolved in normal saline to a concentration of 10 mg/ml as a stock solution and was then diluted to the proper concentration with normal saline. HupA (0.5 mg/kg) or an equal volume of saline was injected IP 30 min after the first injury and continued once per day for 30 days (Figure 1). 

***Morris water maze test***

The Morris water maze task was performed to evaluate the ability of spatial learning and memory of the mice, as previously described (20). A white pool (100 cm diameter, 60 cm deep) was filled with water to 29 cm depth. A target platform was positioned 1 cm below the surface of the water in one quadrant of the pool. During hidden and visible platform trials, mice were placed in the tank facing the wall and allowed to swim to find the platform. Each animal was given 60 sec to find the platform and remained on the platform for 10 sec. Mice that failed to mount the platform within the allotted time were guided to the platform by the operator and allowed 10 sec to become acquainted with its location. For probe trials, mice were placed in the tank with the platform removed and given 60 sec to explore the tank. Training test was conducted with four trials per day for five consecutive days(18). On the sixth and seventh days, a probe test was performed in which the hidden platform was removed and the time spent in the target quadrant within a 60 sec period was used to evaluate the spatial memory ability of the mice. After the behavior test, mice were sacrificed, then the brains were immediately removed from the skull for further experiments (Figure 1).

***Brain water content***

Brain water content was quantified as previously described (8). It was measured by the wet-dry weight ration method (18,21). Animals were anesthetized with sodium pentobarbital (50 mg/kg; IP) and the brains were quickly dissected at 24 hr following TBI. The cerebellum and brainstem were discarded, while the left cortical tissue was harvested. The wet weight of each cortical tissue was measured, then the sections were dried at 80 °C for 72 hr to obtain the dry weight. The brain water content (%) was calculated using the following formula = [(wet weight-dry weight)/wet weight] × 100% (22).

***Brain tissue harvest***

Animals were deeply anesthetized and perfused transcardially with ice-cold saline. The brains were dissected and the hemispheres were divided. The hippocampus and cortex were dissected and proteins were extracted for Western blot analysis (four mice per group) and stored at −80 °C before use. Other brain tissues were fixed in 4% paraformaldehyde in PBS to be used for HE staining (Figure 1).

***H&E staining***

The brain was irrigated with 4 % paraformaldehyde and immersed in a fixative for 24–72 hr at room temperature, followed by the routine dehydration procedure, embedded in paraffin wax, cut into 6 μm brain sections by a rotative microtome, and stained with hematoxylin-eosin (H&E). Then, the sections were photographed under a light microscope. 

***Measurements of malondialdehyde (MDA) level and glutathione peroxidase (GPx) activity***

The MDA content and GPx activity were measured according to a previous study(19). The levels of MDA and GPx were evaluated using a commercially available kit (Nanjing Jiancheng Bioengineering Institute, Nanjing, China). The level of MDA was expressed as nmol/mg protein. Tissue samples were homogenized in ice-cold phosphate buffer saline (PBS) and centrifuged at 12,000 rpm for 15 min at 4 °C. The concentrations of total protein were determined by the Bradford method. The supernatants were used to measure the content of MDA and the activity of GPx using a spectrophotometer. The activity of GPx was expressed as U/mg protein.

***Western blot analysis***

Animals were sacrificed 1 month after the last injury to determine expression of Nrf2, HO-1, and synaptophysin (SYP) (n=10). An Enhanced BCA Protein Assay Kit (Beyotime, Jiangsu, China) was used to determine the protein concentration. The brain tissues were lysed in RIPA buffer and were centrifuged at12,000 rpm for 20 min at 4 °C, and supernatants were collected. An equal amount (50 μg) of the protein samples were separated by 4%–15% SDS polyacrylamide gels (Bio-Rad, CA), then transferred to a polyvinylidene fluoride (PVDF) membrane. The membranes were blocked with 5% fat-free milk for 1 hr and then incubated with primary antibodies Nrf2 (1:1000), HO-1 (1:1000), SYP (1:1000), or β-actin (1:5000) (Abcam, Cambridge, MA, USA) overnight at 4 °C. Then, the membranes were incubated for 1 hr at room temperature with horseradish peroxidase-conjugated secondary antibodies (1:2500, Proteintech, Rosemont, IL). The membranes were washed three times in Tris Buffered Saline with Tween (TBST), protein bands were detected with enhanced chemiluminescence (BioRad, Hercules, CA, USA). Quantification of immunoreactive bands was quantified by Image J software. The density of each sample was normalized to the density of β-actin.

***mRNA extraction and quantitative real-time polymerase chain reaction analysis***

Total RNAs were extracted from the brain samples using IllustraRNAspin Mini Kit (GE healthcare life science, Pittsburgh, PA, USA). Complementary DNA (cDNA) was synthesized from one microgram of total RNA using iScript RT-qPCR Kit (BIO-RAD, Hercules, CA, USA). StepOne™ from Applied Biosystems was used for quantitative real-time polymerase chain reaction analysis. The primers used in the study were as follows: 

Primer HO-1Forward: 

5’-GGGTGATAGAAGAGGCCAGACT-3’

Primer HO-1 Reverse: 

5’-AGCTCCTGCAACTCCTCAAAGA-3’

Primer SYP Forward: 

5’-TGTGCCAACAAGACGGAGAG-3’

Primer SYP Reverse: 5’-TTTAACGCAGGAGGGTGCAT-3’

Primer Nrf2 Forward: 5′-GAACGAGCTTCGCTGAG-3’

Primer Nrf2 Reverse: 5’- ATGACCTTGGGGTGGATG-3’

Primer GAPDH Forward: 

5’-GGAGAGCTAGGGGCAAGC-3’

Primer GAPDH Reverse: 5’-TCGTCCCGTAGACAAAAT

GG-3’

Amplifications were carried out in triplicate and the relative expression of target genes was determined by the ΔΔCT method. Quantification of targeting genes measured was normalized to that of GAPDH in the same sample.

***Statistical analysis***

All statistical analyses were carried out with SPSS Statistical Software (version 16). The quantitative data were expressed as mean ± SD with *P*<0.05 being considered statistically significant. The analysis of one-way ANOVA was used to compare differences among multiple groups.

**Figure 2:**
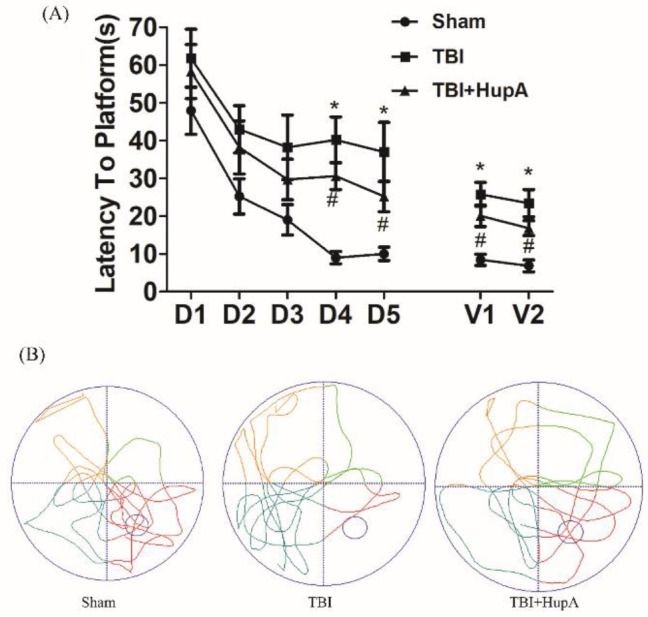
Effects of HupA on memory function in each group. Learning and memory ability of mice were assessed by the Morris water maze test for 5 consecutive days. (A) Escape latency during the training phase (D1–D5) and the time spent in the target quadrant (V1–V2) during visible platform trials. (B) Representative swimming path tracing of searching for the target quadrant on the fifth day. Data are expressed as mean ± SD. (n = 20 mice). * *P*<0.05 vs sham, # *P*<0.05 vs TBI

Huperzine A; TBI: Traumatic brain injury

**Figure 3:**
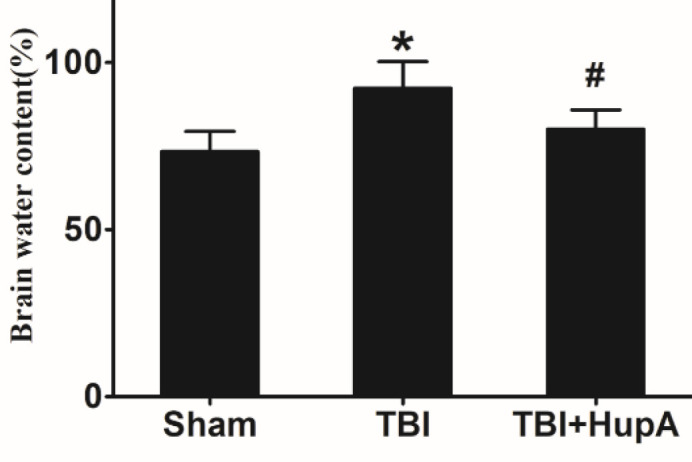
Effects of HupA on brain water content in mice 24 hr after the last injury. Data are expressed as mean±SD (n=5 mice). * *P*<0.05 vs sham, # *P*<0.05 vs TBI

Huperzine A; TBI: Traumatic brain injury

**Figure 4:**
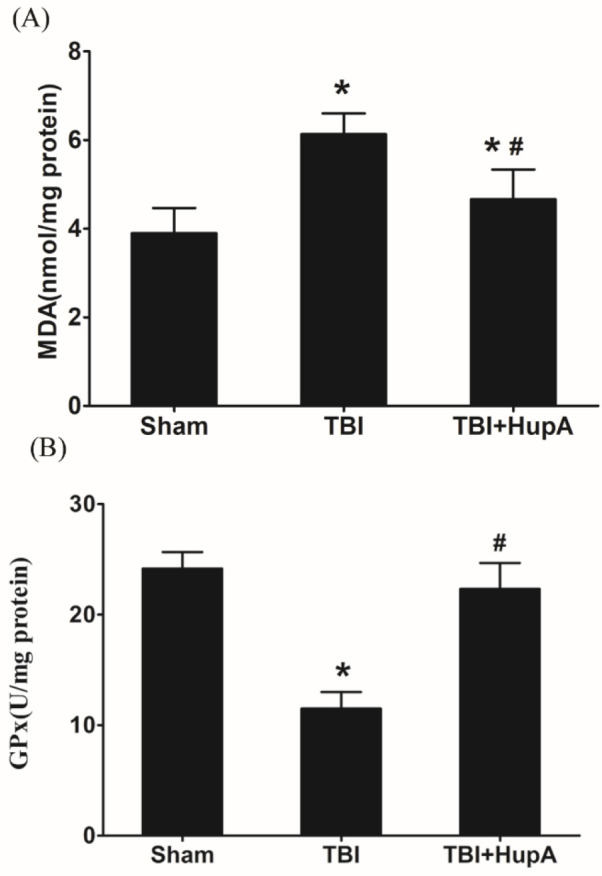
HupA alleviated oxidative stress caused by TBI. (A) Measurement of malondialdehyde (MDA) levels. (B) GPx activity. Data are expressed as mean±SD (n=5 mice). * *P*<0.05 vs sham, # *P*<0.05 vs TBI

Huperzine A; TBI: Traumatic brain injury

**Figure 5:**
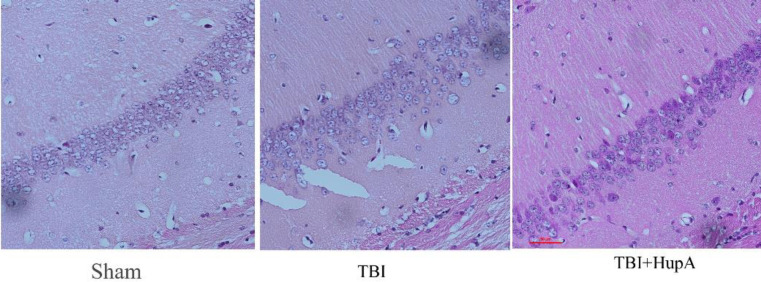
Effects of HupA on the hippocampus morphological changes in TBI mice. Brain sections from mice were stained with hematoxylin and eosin (H&E). (n =3 mice)

Huperzine A; TBI: Traumatic brain injury

**Figure 6:**
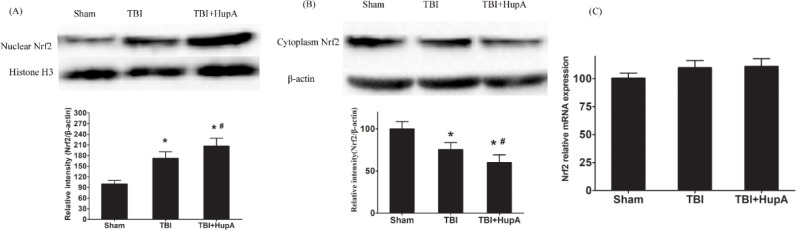
Effects of HupA on Nrf2 mRNA and protein expression. Western blot analy-sis of Nrf2 protein levels and PCR analysis of Nrf2 mRNA expression in each group. (A) Nuclear Nrf2; (B) Cytoplasmic Nrf2; (C) Nrf2 mRNA expression. Data are ex-pressed as mean±SD (n=10 mice). * *P*<0.05 vs sham,# *P*<0.05 vs TBI

Huperzine A; TBI: Traumatic brain injury

**Figure 7:**
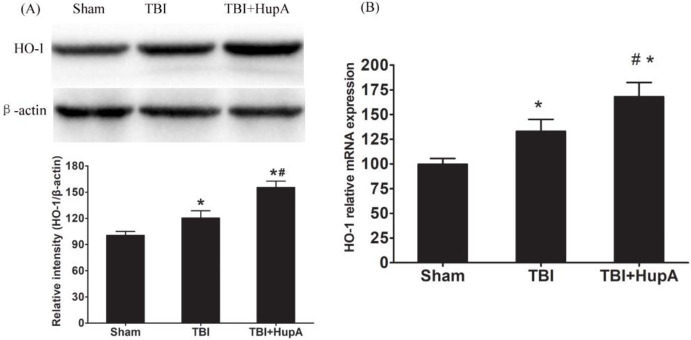
Effects of Hup A on HO-1 mRNA and protein expression. Western blot analysis of HO-1 protein levels and PCR analysis of HO-1 mRNA expression in each group. Data are expressed as mean±SD (n = 10 mice). * *P*<0.05 vs sham, # *P*<0.05 vs TBI

Huperzine A; TBI: Traumatic brain injury

**Figure 8.**
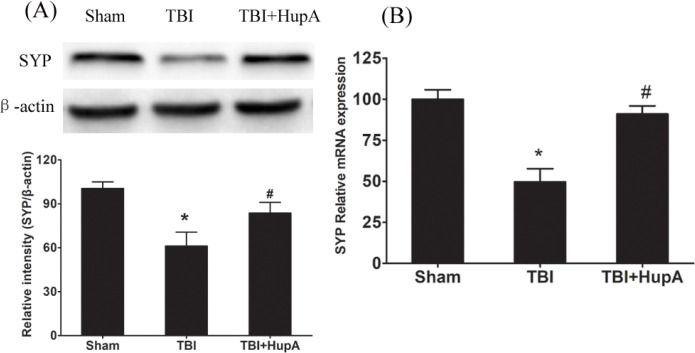
Effects of Hup A on SYP mRNA and protein expression. Western blot analy-sis of SYP protein levels and PCR analysis of SYP mRNA expression in each group. Data are expressed as mean±SD (n = 10 mice). * *P*<0.05 vs sham,#* P*<0.05 vs TBI

Huperzine A; TBI: Traumatic brain injury

## Results

***HupA improved cognitive impairment in TBI mice***

The Morris water maze task was used to evaluate whether HupA improved the cognitive function of TBI mice. Mice were trained to find the hidden platform, after the training session, we observed the time of arriving at the hidden platform. The less time it takes for mice to reach the hidden platform, the better the spatial learning and memory function is. Mice were tested on day 31 after the first injury. As shown by the Morris water maze test, compared with the sham group, TBI animals took more time to arrive at the hidden platform. However, compared with the vehicle-treated TBI group, HupA treated mice showed a trend to spend less time to arrive at the platform (Figure 2A). Moreover, TBI mice passed the former platform location significantly fewer times than the sham group in the probe trial on the last day of testing. TBI mice treated with Hup A passed the former platform location significantly more times than TBI mice treated with vehicle (Figure 2B). Overall, these behavioral tests indicate that TBI mice show significant cognitive deficits in spatial learning and memory function, and HupA treatment improves spatial learning and memory function in TBI mice.

***HupA reduced brain edema in TBI mice***

Brain edema after TBI is the main cause of post-traumatic intracranial hypertension. Considering the brain edema is obvious in the acute stage after TBI, 24 hr after the completion of the model was selected to evaluate the effect of HupA on brain edema after TBI. The percentage of brain water content in the TBI group increased significantly compared with the sham group (*P*=0.0298) and HupA treatment significantly decreased the brain water content compared with the vehicle-treated TBI group (Figure 3).

***HupA reduced oxidative stress in the cortex after TBI***

To investigate the effect of HupA on oxidative stress after TBI, the levels of MDA and GPx activity in the brain tissue were measured. Figure 4A showed that MDA was increased in the TBI groups compared with the sham group (*P*=0.0142); and treatment of HupA after TBI significantly decreased the generation of MDA compared with vehicle-treated injured mice. Conversely, GPx activities were decreased in the TBI group compared with the sham group (*P*=0.0297). However, treatment with HupA partially rescued GPx activity after TBI (Figure 4B). These data suggest that HupA alleviates oxidative stress and enhances anti-oxidant enzyme activity after TBI.

***HupA ameliorated the brain morphological changes in TBI mice***

To determine effects of HupA on brain morphological changes after TBI, we conducted H&E staining on brain sections. The hippocampus CA1 areas were examined using a microscope. Hippocampus CA1 neurons of the sham group had no obvious pathological changes. The neurons were close, the dye was clear, and the cell shapes were normal. In the TBI group, the cells were sparsely arranged, cellular structures were unclear, cytoplasmic vacuolar changes were observed, and nerve-fibers reduced. HupA administration caused marked mitigation of these changes. Therefore, a high degree of cellular integrity was evident in HupA groups (Figure 5).

***HupA promoted Nrf2 nuclear translocation***

Nrf2 plays a key role in the protection of cells and tissues from oxidative damage via up-regulation of anti-oxidant enzymes such as heme oxygenase-1 and peroxiredoxin 1 (23). We further investigated whether Nrf2 was changed after HupA treatment in the TBI mice. Firstly, we examined the expression of Nrf2 by Western blot. The results showed that Nrf2 protein levels in nuclear fraction were increased significantly in both TBI groups compared with the sham group (*P*=0.0006); they were even higher in HupA-treated TBI mice (Figure 6). These results suggest that HupA may activate Nrf2. We further investigated the downstream gene expression of Nrf2. As shown in Figure 7, the mRNA and protein levels of HO-1 were up-regulated after TBI. Moreover, HupA treatment further enhanced the mRNA and protein levels of HO-1. These data indicate that HupA promotes Nrf2 translocation from cytoplasm to the nucleus, thereby obtaining the elevated binding ability to the downstream gene expression.

***Effect of HupA on SYP***

It is widely recognized that synaptic dysfunction is one of the major bases of neurological dysfunction after TBI (24). SYP mRNA expression was significantly higher in the HupA treated TBI group than in the TBI mice. SYP protein content was examined by Western blot. As shown in Figure 8, SYP protein expression was significantly decreased in the TBI group compared with the control group (*P*=0.0021). As expected, HupA treatment increased SYP expression after TBI. These data suggest that HupA may improve cognitive function by up-regulating synaptic proteins.

## Discussion

In this study, we found that HupA treatment significantly attenuated brain edema, decreased level of MDA, increased GPx activity, and resulted in the activation of the Nrf2 pathway in TBI mice. HupA may be used to suppress oxidative injury and improve behavioral outcomes.

Cognitive decline is one of the most obvious symptoms of TBI (25). Spatial cognition was shown impaired in TBI mice through measuring time spent to find the platform. It is consistent with previous reports (26). Spatial learning and memory after TBI in mice were evaluated by the Morris water maze test. We found that the HupA group spent less time finding the platform compared with the vehicle group, suggesting that HupA alleviated cognitive impairment after TBI. 

Oxidative stress is one of the major mechanisms of secondary injury followed by TBI (27, 28). Evidence strongly suggests that excessive reactive oxygen species (ROS) are generated by the imbalance between free radicals and anti-oxidants which propagates secondary injury after TBI (26). With the continuous production of ROS, protein nitration, lipid peroxidation, and DNA damage may arise subsequently, at the same time anti-oxidant enzymes may be gradually exhausted (29). Balanced cellular oxidants and anti-oxidants are important to maintain redox homeostasis. Accumulating evidence indicates that HupA can inhibit the production of ROS. HupA has been reported to attenuate oxidative glutamate toxicity in murine hippocampal HT22 cells by activating the PI3K/Akt/mTOR signaling pathway (30) and decreased ROS generation as well as oxidative damage in D-galactose-treated rats (31). In addition, HupA could significantly decrease ROS production by X-ray radiation in NIH3T3 cells (11). In the present study, HupA showed antioxidative effects in TBI, including increased GPx activities and decreased MDA content, in line with previous studies.

Nrf2 is a regulator of endogenous anti-oxidant defense, which regulates the expression of various anti-oxidant genes (32). Under physiological conditions, Nrf2 is mainly located in the cytoplasm and Nrf2 translocates into the nucleus to bind to the anti-oxidant response element (ARE) when oxidative stress is inducted. Nrf2 promotes the expression of anti-oxidant genes including HO-1, quinone oxidoreductase (NQO1), and GST. Anti-oxidant enzymes, such as SOD, GPx, and CAT, protect cells from oxidative damage (33). As described in our results, treatment with HupA effectively increased the activities of several anti-oxidant enzymes in brain tissues. Emerging evidence indicates that Nrf2 plays an important protective role in TBI, and Nrf2 activation ameliorates TBI-induced damage. Our data further showed that HupA promoted translocation of Nrf2 from the cytoplasm to the nucleus. The results suggested that HupA inhibited oxidative stress by increasing endogenous Nrf2 activity. The evidence indicates that the anti-oxidant effect of the Nrf2-ARE pathway after TBI depends on up-regulated Phase II detoxifying enzymes, such as NQO1 and HO-1(34-36). To clarify whether the Nrf2-ARE pathway participated in the neuroprotective effects of HupA, the expression of HO-1 was evaluated by Western blot and the results showed that HupA treatment further enhanced the mRNA and protein levels of HO-1 after TBI. SYP is a synaptic vesicle glycoprotein that is widely expressed on presynaptic membranes and strongly associated with synaptic plasticity (37). 

The amount of SYP expression reflects the level of synaptic function and synaptic transmission (38). Elevated SYP levels have a positive effect on consolidation of learning and memory after injury (39).

Our present findings demonstrate HupA significantly enhanced expression of SYP in brain tissue after TBI. These changes were accompanied by improvements in neurological function. Taken together, these results indicate that HupA attenuates TBI-induced cognitive deficits via up-regulation of SYP. 

Collectively, the present results suggest that HupA has neuroprotective properties against TBI-related damage. HupA may be an effective neuroprotectant as a pharmacological intervention for TBI treatment.

## Authors’ Contributions

ZM and BS Study conception and design; YH and HY data processing, collection, performing experiments; YH Data analysis and draft manuscript preparation; BS Analysis and interpretation of results; HY Draft manuscript preparation, visualization; ZM Critical revision of the paper; QS Supervision of the research; ZM Funding acquisition; ZM, YH, HY, QS, and BS Final approval of the version to be published.

## Funding Information

This work was supported by a grant from the Project of Traditional Chinese Medicine Bureau of Guangdong Province (20212136).

## Conflictes of Interest

No competing ﬁnancial interests exist.
